# Azithromycin in Dentistry: From Systemic Antibiotic to a Candidate for Local Therapeutic Delivery

**DOI:** 10.3390/pharmaceutics18081004

**Published:** 2026-08-14

**Authors:** Jakub Kwiatek, Magdalena Paczkowska-Walendowska, Judyta Cielecka-Piontek

**Affiliations:** 1 Kwiatek Dental Clinic Sp. z o.o., Kordeckiego 22, 60-144 Poznan, Poland; jakubkwiatek@klinikakwiatek.pl; 2 Department of Pharmacognosy and Biomaterials, Poznan University of Medical Sciences, Rokietnicka 3, 60-806 Poznan, Poland; jpiontek@ump.edu.pl; 3 Science-Bridge Sp. z o.o., Chociszewskiego 24/8, 60-258 Poznan, Poland

**Keywords:** azithromycin, local drug delivery, dentistry, periodontitis, regenerative dentistry, biofilm

## Abstract

Azithromycin is widely used in dentistry as a systemic antibiotic, particularly for odontogenic infections and as an alternative in patients with β-lactam hypersensitivity. Beyond its antimicrobial activity, azithromycin possesses unique pharmacokinetic, anti-inflammatory, immunomodulatory, and anti-biofilm properties. Together with growing concerns regarding antimicrobial resistance and antibiotic stewardship, these characteristics have stimulated interest in local drug-delivery strategies that may reduce systemic antibiotic exposure while maintaining therapeutic efficacy. This narrative review evaluates the rationale, potential clinical applications, and current evidence supporting local azithromycin delivery in dentistry. The available literature on azithromycin pharmacology, systemic dental use, immunomodulatory mechanisms, biofilm-related effects, local drug-delivery systems, safety, and regulatory considerations was critically reviewed. Current evidence suggests that locally delivered azithromycin may achieve high drug concentrations at the target site, enhance anti-biofilm activity, modulate local inflammation, and minimize systemic exposure. Potential applications include periodontitis, peri-implant diseases, persistent endodontic infections, oral surgery, and regenerative procedures such as bone augmentation and maxillary sinus floor elevation. Emerging delivery platforms, such as hydrogels, thermoresponsive gels, nanoparticles, and chitosan-based systems, further support the feasibility of this approach. Experimental findings also indicate that azithromycin may inhibit osteoclast activity, suggesting additional benefits for bone preservation and regenerative healing. Despite these promising findings, current evidence remains limited and is derived mainly from preclinical studies and small clinical investigations. Further translational research and well-designed randomized controlled trials are needed to establish the safety, efficacy, and optimal clinical role of locally delivered azithromycin in evidence-based dental practice.

## 1. Introduction

Oral infectious diseases are among the most prevalent chronic conditions worldwide and represent a major public health challenge. According to the Global Burden of Disease Study, untreated oral diseases affect approximately 3.5 billion people, while severe periodontitis affects around 1 billion individuals worldwide, making it one of the most common chronic inflammatory diseases [1]. Although oral infections are rarely directly associated with mortality, they are a significant cause of pain, tooth loss, impaired mastication, reduced quality of life, and substantial healthcare expenditure [2]. In severe cases, odontogenic infections may spread to deep fascial spaces, resulting in life-threatening complications requiring hospitalization [3]. Moreover, increasing evidence links chronic oral infections with systemic conditions, including cardiovascular diseases, diabetes mellitus, and adverse pregnancy outcomes [2].

Antibiotics remain an integral component of dental practice, particularly in the management of acute odontogenic infections, selected periodontal and peri-implant diseases, and as prophylaxis in specific surgical and medically compromised patient populations [4]. However, dentistry accounts for a substantial share of outpatient antibiotic prescriptions, and growing evidence indicates that a significant proportion of these prescriptions are unnecessary or suboptimal [5]. This overuse has direct implications for the global rise in antimicrobial resistance, prompting increased pressure to reassess traditional antibiotic strategies and to explore more targeted, stewardship-oriented approaches in dental care [6].

**Figure 1:**
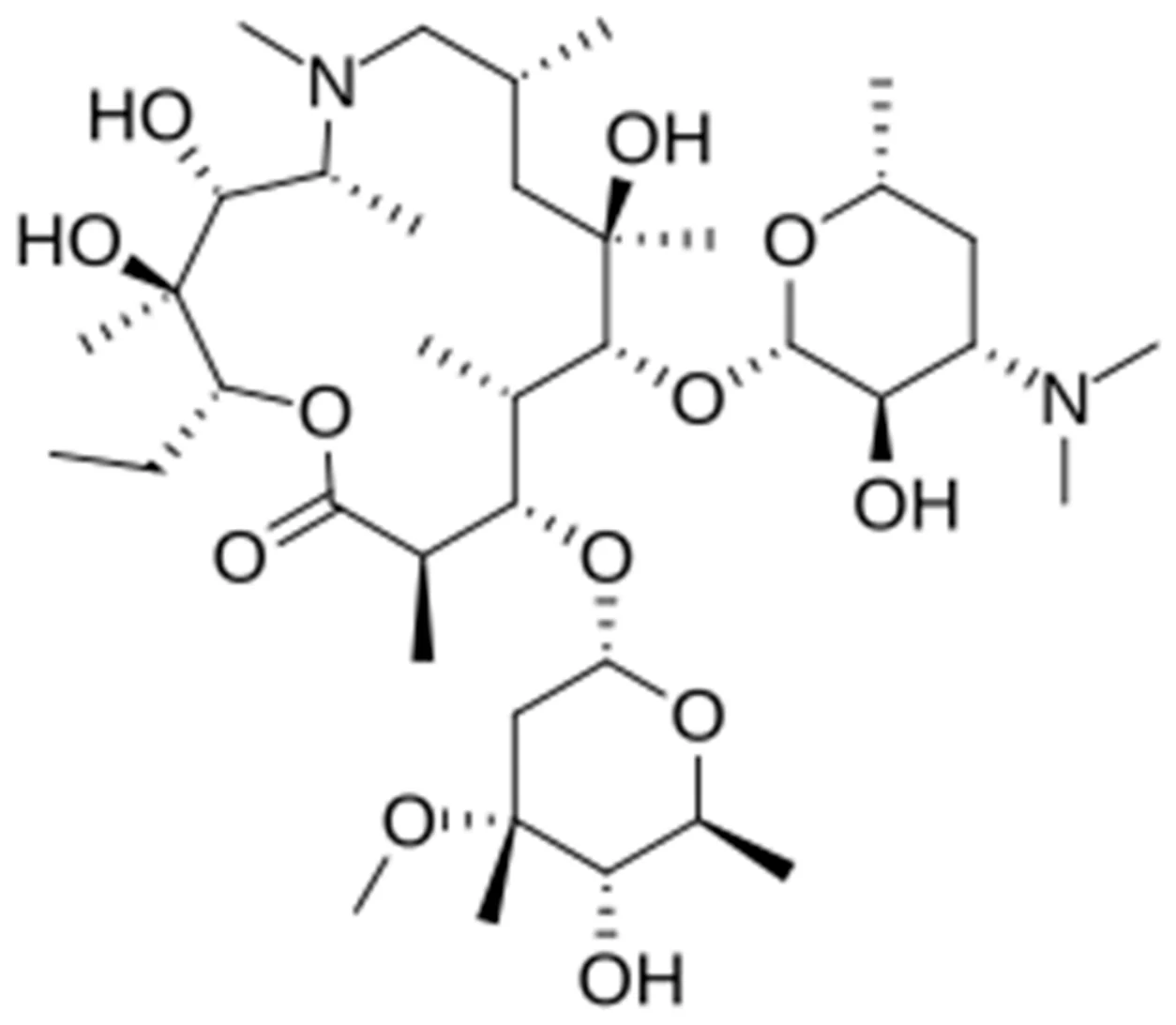
Chemical structure of azithromycin.

Azithromycin (Figure 1), AZC, a second-generation macrolide antibiotic belonging to the azalide subclass, is commonly prescribed in dentistry, especially for patients with hypersensitivity to beta-lactam antibiotics [7]. Its popularity stems from a combination of broad antimicrobial activity, favorable tolerability, convenient dosing schedules, and excellent tissue penetration [8]. Most existing dental literature and clinical guidelines, however, address azithromycin almost exclusively as a systemically administered antibiotic, focusing on its role as an alternative to penicillins or clindamycin for acute infections [9,10].

Beyond its antibacterial properties, azithromycin exhibits well-documented anti-inflammatory and immunomodulatory effects, including modulation of cytokine production, neutrophil function, macrophage polarization, and biofilm behavior [11]. These properties have been extensively studied in respiratory medicine and dermatology, but have received comparatively limited and fragmented attention in the dental literature [12,13]. Importantly, many chronic oral diseases, such as periodontitis, peri-implantitis, and persistent endodontic infections, are driven not only by microbial challenge but also by dysregulated host immune responses [14]. This dual pathophysiology raises the question of whether azithromycin’s therapeutic value in dentistry extends beyond conventional systemic antimicrobial use.

Several review articles have addressed antibiotic use in dentistry broadly, local drug delivery systems in periodontology, or the pharmacology of macrolides in general [15,16]. However, to date, no comprehensive review has systematically repositioned azithromycin as a candidate for local application in dentistry by integrating its pharmacokinetic characteristics, immunomodulatory effects, emerging formulation strategies, safety considerations, and regulatory challenges into a single, coherent framework. Existing reviews that mention azithromycin in local delivery contexts typically do so briefly, often limited to individual clinical trials or formulation studies, and fail to synthesize the broader translational implications critically.

The novelty of the present review lies in its conceptual shift: rather than treating azithromycin solely as a systemic substitute antibiotic, this work evaluates azithromycin as a multifunctional agent with potential local therapeutic relevance in dentistry. This review uniquely integrates evidence from systemic dental use, immunobiology, biofilm science, and modern drug-delivery technologies to explore whether local azithromycin administration could represent a rational adjunct or alternative to systemic antibiotic therapy in selected clinical scenarios.

Furthermore, this review explicitly situates local azithromycin use within the context of antibiotic stewardship, regulatory constraints, and patient-specific therapeutic decision-making. By critically examining not only potential benefits but also safety concerns, resistance risks, and research gaps, this work aims to provide a balanced and clinically relevant perspective that can inform both future research and cautious clinical translation.

The aim of this review is therefore not to advocate premature clinical adoption, but to offer a structured, evidence-based analysis of azithromycin’s evolving role in dentistry, with particular emphasis on its potential for local application. By identifying areas of promise and unresolved challenges, this review seeks to advance the development of more targeted, effective, and responsible antibiotic strategies in modern dental practice.

## 2. Literature Search Strategy

The literature included in this narrative review was identified through searches of PubMed, Scopus, and Web of Science databases up to June 2026 using combinations of the keywords “azithromycin”, “dentistry”, “local drug delivery”, “periodontitis”, “peri-implantitis”, “oral surgery”, “endodontics”, “hydrogels”, “nanoparticles”, and “electrospinning”. Priority was given to systematic reviews, randomized clinical trials, translational studies, and recent publications, while selected experimental studies were included to discuss emerging therapeutic concepts.

## 3. Pharmacological Profile of Azithromycin in Dentistry

Azithromycin is a semi-synthetic azalide antibiotic derived from erythromycin. The incorporation of a methyl-substituted nitrogen atom into the lactone ring distinguishes azalides from classical macrolides and enhances acid stability, tissue penetration, and intracellular accumulation. These structural modifications improve its pharmacokinetic profile while reducing gastrointestinal adverse effects compared with earlier macrolides [17,18,19].

Azithromycin exerts its antibacterial activity by binding to the 50S ribosomal subunit, thereby inhibiting bacterial protein synthesis. It is primarily bacteriostatic but may exhibit bactericidal activity at higher concentrations. The antibiotic demonstrates activity against a broad spectrum of Gram-positive and Gram-negative bacteria, as well as atypical pathogens, including microorganisms commonly implicated in oral infections, such as *Streptococcus* spp., *Aggregatibacter actinomycetemcomitans*, and *Porphyromonas gingivalis* [20,21].

### 3.1. Pharmacokinetic Properties Relevant to Dentistry

The pharmacokinetic profile of azithromycin represents one of its major therapeutic advantages in dental practice. Following oral administration, azithromycin is rapidly absorbed, with an oral bioavailability of approximately 37%, and exhibits an exceptionally large apparent volume of distribution (approximately 31 L/kg), reflecting extensive penetration into peripheral tissues [22]. Tissue concentrations may exceed plasma concentrations by more than 100-fold owing to active uptake by fibroblasts, epithelial cells, macrophages, and neutrophils. These cells subsequently release the drug at sites of infection and inflammation, enabling sustained local antimicrobial activity.

Azithromycin is characterized by a prolonged terminal elimination half-life of approximately 60–70 h, allowing once-daily administration and short treatment regimens while maintaining therapeutic concentrations for several days after the final dose. In addition, the drug exhibits a pronounced post-antibiotic effect, which further contributes to its prolonged antimicrobial activity. Importantly for dentistry, azithromycin achieves high concentrations in gingival crevicular fluid, saliva, periodontal tissues, and inflamed oral mucosa, providing a strong pharmacokinetic rationale for both systemic administration and the development of local drug-delivery systems [23]. These characteristics are particularly advantageous in chronic oral infections, where intracellular bacterial reservoirs and limited tissue penetration often compromise the efficacy of conventional antibiotics.

### 3.2. Current Systemic Use of Azithromycin in Dentistry

Systemic antibiotic therapy remains an important adjunct in the management of dental infections presenting with systemic involvement, rapid progression, or an increased risk of dissemination [24,25]. Owing to its broad antimicrobial spectrum, favorable pharmacokinetic profile, and good tolerability, azithromycin is widely prescribed in dentistry, particularly for patients with documented hypersensitivity to β-lactam antibiotics [26].

The principal indications include acute odontogenic infections accompanied by fever, lymphadenopathy, or facial swelling, selected periodontal and peri-implant infections refractory to mechanical therapy, and persistent endodontic infections when immediate conventional treatment is not feasible [26,27,28]. Clinical studies have demonstrated that azithromycin is as effective as amoxicillin and clindamycin in selected dental infections [29]. In periodontal therapy, its adjunctive use with scaling and root planing has been associated with reductions in probing pocket depth, clinical attachment loss, and inflammatory parameters [29]. The drug’s high tissue penetration and intracellular accumulation contribute substantially to these clinical outcomes by maintaining therapeutic concentrations within periodontal tissues and gingival crevicular fluid [23].

An additional advantage of azithromycin is its simplified dosing schedule, typically once daily for three to five days, which improves patient adherence compared with antibiotics requiring prolonged or multiple-dose regimens [30,31]. Nevertheless, systemic administration is associated with several limitations. Gastrointestinal disturbances remain the most common adverse events, while QT interval prolongation and cardiac arrhythmias may occur in susceptible patients [32,33]. Furthermore, increasing macrolide resistance among oral and non-oral microorganisms represents an important public health concern [34]. Consequently, contemporary guidelines recommend systemic azithromycin only when clearly indicated and always as an adjunct to definitive mechanical or surgical treatment, reinforcing the need for alternative strategies to reduce unnecessary systemic antibiotic exposure.

### 3.3. Anti-Inflammatory and Immunomodulatory Properties

Beyond its antimicrobial activity, azithromycin possesses well-documented anti-inflammatory and immunomodulatory properties that substantially contribute to its therapeutic potential. These effects have been extensively investigated in respiratory and dermatological diseases and are increasingly recognized as clinically relevant in oral and periodontal disorders [35].

**Figure 2:**
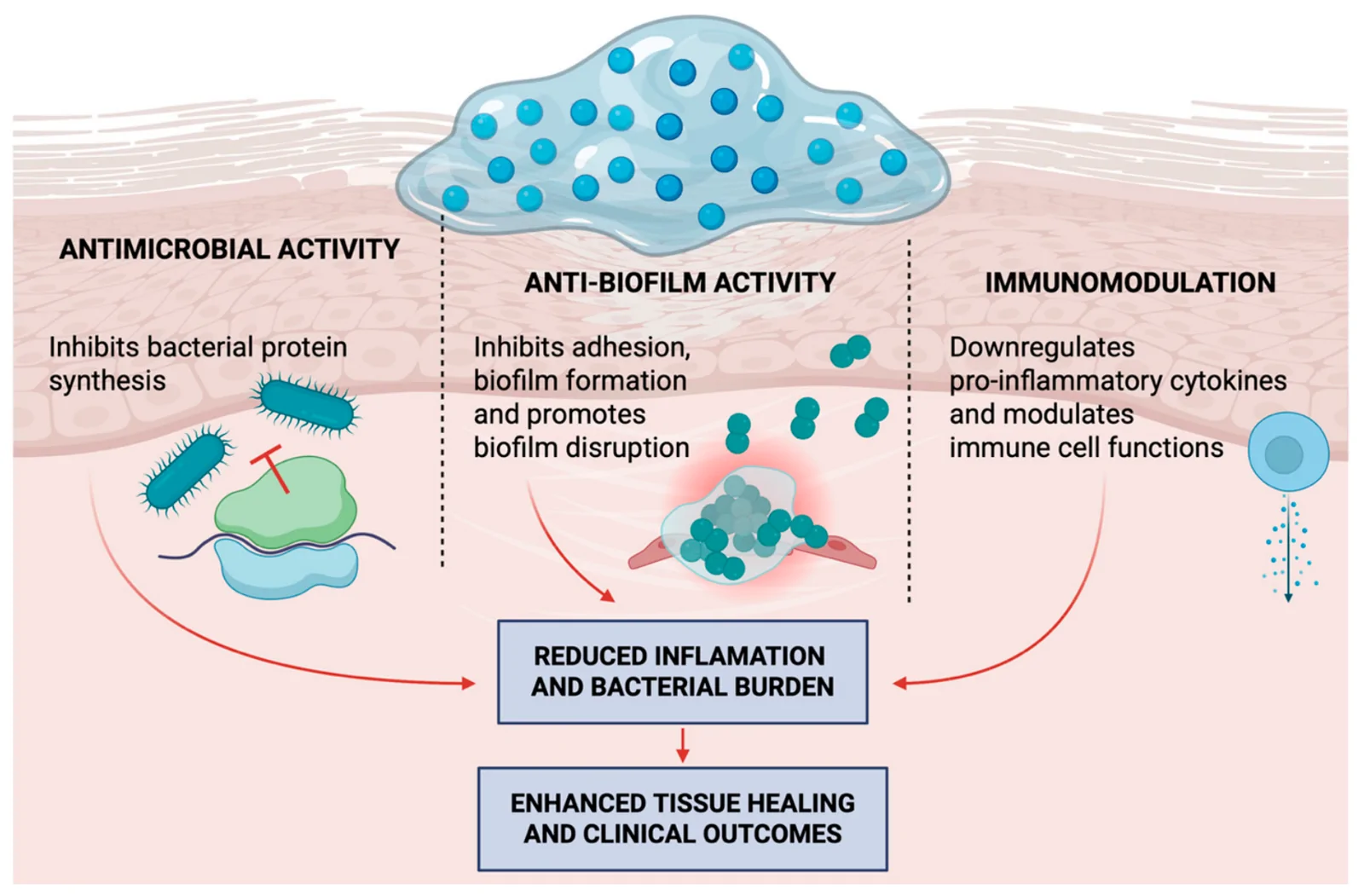
Mechanisms of action of locally delivered azithromycin.

Azithromycin attenuates inflammatory responses by suppressing the production of major pro-inflammatory cytokines, including interleukin-1β, interleukin-6, and tumor necrosis factor-α, thereby limiting cytokine-mediated tissue destruction associated with chronic oral infections [36,37]. At the cellular level, it modulates innate immune responses by reducing neutrophil chemotaxis, oxidative burst, and the release of tissue-destructive enzymes, while promoting neutrophil apoptosis and clearance [38]. Moreover, azithromycin induces macrophage polarization toward an anti-inflammatory phenotype associated with tissue repair and regeneration [39] (Figure 2).

In addition to its immunomodulatory activity, azithromycin interferes with bacterial biofilm formation and maturation by affecting quorum-sensing pathways and bacterial virulence factors [40]. These properties are particularly relevant in periodontitis and peri-implantitis, where mature biofilms are highly resistant to both host immune responses and conventional antimicrobial therapy [41]. Notably, many of these immunomodulatory effects occur at tissue concentrations achievable during routine treatment and appear to be independent of the drug’s direct antibacterial activity [42]. Consequently, azithromycin may simultaneously target both the microbial component of disease and the dysregulated host inflammatory response.

This dual mechanism of action distinguishes azithromycin from many conventional antibiotics used in dentistry and provides a strong biological rationale for its further development as a locally delivered therapeutic agent. By combining antimicrobial, anti-biofilm, and immunomodulatory effects while minimizing systemic exposure, local azithromycin formulations may represent a promising strategy for the management of chronic oral inflammatory diseases [43].

## 4. Rationale for Local Use of Azithromycin in Dentistry

The increasing emphasis on antibiotic stewardship and minimally invasive therapeutic approaches has heightened interest in local drug-delivery systems in dentistry [44]. Local administration enables the delivery of high concentrations of antimicrobial agents directly to the site of infection while minimizing systemic exposure, thereby reducing the risk of systemic adverse effects and the selection of resistant bacterial strains. This strategy is particularly relevant in the management of localized oral infections, where mechanical therapy alone may be insufficient to control microbial and inflammatory processes fully [45].

Systemic antibiotic therapy in dentistry is often limited by restrictive guidelines [46] but also suboptimal drug concentrations at the target site, patient-related contraindications, and the potential for unwanted systemic effects [47]. In contrast, local drug delivery enables targeted therapy within periodontal pockets, peri-implant tissues, root canals, or surgical sites, achieving concentrations that exceed the minimum inhibitory concentration against key oral pathogens. Such high local concentrations may enhance antimicrobial efficacy, particularly against biofilm-associated bacteria that are inherently more resistant to systemic antibiotics [48].

Azithromycin possesses several pharmacological characteristics that make it a promising candidate for local application. Its strong tissue affinity and intracellular accumulation suggest that sustained local retention may be achievable when delivered directly to oral tissues. Moreover, azithromycin’s documented anti-inflammatory and immunomodulatory effects may be particularly beneficial in chronic inflammatory dental conditions, where host-mediated tissue destruction plays a central role. By modulating cytokine production and immune cell activity locally, azithromycin may contribute not only to microbial control but also to the resolution of inflammation and the promotion of tissue healing [22].

Another important consideration is azithromycin’s activity against oral biofilms. Biofilm-associated infections, such as periodontitis, peri-implantitis, and persistent endodontic infections, represent a major therapeutic challenge due to reduced antibiotic penetration and altered bacterial metabolism. Local delivery of azithromycin may help overcome these limitations by maintaining prolonged drug exposure at the biofilm–tissue interface, potentially enhancing its antibiofilm and anti-virulence effects when combined with mechanical debridement [49].

From a clinical perspective, local azithromycin application may reduce or eliminate the need for systemic antibiotic therapy in selected cases, thereby supporting responsible prescribing practices. This approach may be particularly advantageous in patients at increased risk of systemic adverse effects, those with complex medical histories, or individuals for whom systemic antibiotics are contraindicated [50]. Additionally, local therapy may improve patient acceptance and adherence by simplifying treatment regimens and minimizing systemic drug intake.

Taken together, these considerations provide a strong rationale for exploring the local use of azithromycin in dentistry. While current evidence remains limited, the convergence of pharmacological properties, biological effects, and clinical needs supports further investigation into local azithromycin delivery as an adjunctive or alternative therapeutic strategy in selected dental indications (Table 1).

## 5. Potential Local Applications of Azithromycin

The local application of azithromycin in dentistry represents a novel therapeutic approach that exploits its antimicrobial and immunomodulatory properties while minimizing systemic exposure. Although clinical evidence remains limited, experimental data and indirect clinical observations support the exploration of azithromycin as a locally delivered adjunct across several dental disciplines.

### 5.1. Periodontology

In periodontology, local drug delivery systems are commonly used as adjuncts to scaling and root planing, particularly in patients with moderate to severe periodontitis or in sites that respond poorly to conventional mechanical therapy [51]. Locally delivered azithromycin may offer advantages in this context due to its activity against key periodontal pathogens, including Gram-negative anaerobes, as well as its capacity to modulate the host inflammatory response.

The combination of antibacterial action and local immunomodulation may contribute to improved clinical outcomes, such as reductions in probing pocket depth, gains in clinical attachment level, and decreased bleeding on probing. Additionally, azithromycin’s ability to penetrate periodontal tissues and accumulate intracellularly suggests it may be effective against bacteria residing within epithelial cells and connective tissues, which are less accessible to mechanical debridement alone [52].

Local administration may be particularly relevant in aggressive or refractory forms of periodontitis, where systemic antibiotic therapy is often considered but carries a higher risk of adverse effects and resistance development [53]. By delivering azithromycin directly into periodontal pockets, therapeutic concentrations can be achieved at diseased sites while avoiding unnecessary systemic exposure [54].

### 5.2. Peri-Implant Diseases

Peri-implant mucositis and peri-implantitis pose significant therapeutic challenges due to the complex topography of implant surfaces and the tenacity of biofilms on titanium or ceramic surfaces. Mechanical decontamination alone is frequently insufficient, and adjunctive antimicrobial therapies are often employed to improve clinical outcomes [55].

Azithromycin’s efficacy against peri-implant-associated microorganisms, combined with its anti-inflammatory and immunomodulatory effects, suggests potential benefits when applied locally to peri-implant tissues [56]. Local delivery may help reduce the inflammatory burden, suppress pathogenic biofilms, and support soft-tissue healing around implants. Furthermore, the anti-inflammatory properties of azithromycin may be particularly advantageous in peri-implant diseases, where excessive host immune responses contribute to bone resorption and tissue destruction.

Although clinical data on locally applied azithromycin in peri-implant therapy are scarce, its pharmacological profile warrants further investigation, particularly when compared with other locally delivered antimicrobials such as chlorhexidine, tetracyclines, or metronidazole [57].

### 5.3. Endodontics

Persistent endodontic infections are often associated with biofilm-forming, antibiotic-resistant microorganisms that are difficult to eradicate using conventional chemomechanical preparation alone. In this context, locally applied antimicrobial agents, including intracanal medicaments, play a critical role in infection control [58].

Azithromycin has demonstrated in vitro activity against several microorganisms implicated in refractory endodontic infections and has shown the ability to penetrate biofilms [59]. Its potential use as an intracanal medicament may therefore be considered, particularly in retreatment cases or in teeth with persistent apical pathology. Additionally, its anti-inflammatory properties may help reduce periapical inflammation and support periapical healing.

However, the compatibility of azithromycin with commonly used endodontic materials, its stability within the root canal environment, and its potential effects on dentin and periapical tissues require further investigation before clinical application can be recommended.

Furthermore, AZC may have a role in endodontic microsurgery, particularly during apicoectomy procedures for persistent apical periodontitis resistant to conventional root canal treatment. Modern endodontic microsurgery, when performed using contemporary microsurgical techniques and biocompatible retrograde filling materials, demonstrates high success rates ranging from approximately 79% to nearly 100%, depending on case selection and follow-up period. In such clinical scenarios, local antimicrobial strategies that reduce residual biofilm activity and modulate postoperative inflammation may further support periapical healing and improve treatment outcomes. Given azithromycin’s antibiofilm and immunomodulatory properties, its future use as a locally delivered adjunct during endodontic surgery warrants further investigation [60].

### 5.4. Regenerative Treatments, Augmentations, and Maxillary Sinus Lifts

Bone augmentation procedures and maxillary sinus floor elevation are widely performed in contemporary implant dentistry to enable implant placement in patients with insufficient alveolar bone volume [61]. These procedures demonstrate high long-term implant survival and clinical predictability when modern regenerative techniques are applied. However, regenerative procedures remain associated with risks of postoperative infection, inflammatory complications, graft contamination, membrane exposure, and impaired healing, especially in extensive grafting procedures or in medically compromised patients [62,63].

Systemic antibiotic prophylaxis is therefore commonly used in regenerative and implant surgery, although the optimal protocol and duration remain controversial [64]. Current evidence suggests that antibiotic therapy may be particularly beneficial in high-risk situations, including membrane perforation, extensive lateral sinus augmentation, or compromised systemic conditions [62].

In this context, local antimicrobial strategies that deliver high drug concentrations directly to the surgical site while minimizing systemic exposure may be an attractive adjunctive approach. Such localized therapy may be especially valuable in guided bone regeneration and sinus augmentation procedures, where prolonged healing periods and biomaterial exposure create favorable conditions for bacterial colonization and biofilm formation [65].

Azithromycin may be of particular interest in regenerative oral surgery due to its combined antimicrobial, anti-biofilm, and immunomodulatory properties. In addition to its activity against oral pathogens, azithromycin has been shown to modulate inflammatory cytokine expression and support wound healing, which may be advantageous for graft integration and postoperative tissue regeneration [42,60]. Furthermore, its ability to accumulate in soft and hard tissues suggests that sustained local activity may be achievable within grafted areas and peri-implant tissues [22].

Recent investigations comparing azithromycin and amoxicillin in implant-related surgical procedures have highlighted growing interest in azithromycin as a potential alternative perioperative antimicrobial strategy due to its prolonged tissue retention and anti-inflammatory effects [66,67]. However, evidence regarding the local application of azithromycin in bone grafting and sinus augmentation procedures remains limited, and further studies are required to establish optimal formulations, dosing strategies, and long-term clinical outcomes.

### 5.5. Oral Surgery and Wound Healing

Local application of azithromycin in oral surgical settings, such as extraction sockets or postoperative wounds, may reduce the risk of infection and control postoperative inflammation. Surgical trauma induces an inflammatory response that, if excessive, may delay healing or predispose to complications such as alveolar osteitis or surgical site infection [68].

By delivering azithromycin locally, high antimicrobial concentrations can be achieved at the surgical site while simultaneously modulating inflammatory processes. Its immunomodulatory effects may promote a more favorable healing environment by limiting excessive cytokine release and supporting tissue regeneration. This approach may be particularly beneficial in patients with compromised healing capacity or in procedures associated with a higher risk of postoperative infection [42,69].

Despite these theoretical advantages, clinical evidence supporting the routine local use of azithromycin in oral surgery remains limited. Well-designed clinical trials are necessary to evaluate their safety, efficacy, and optimal delivery methods in surgical applications.

Surgical procedures in the oral cavity are inherently associated with disruption of tissue continuity, which may create an entry point for microorganisms and increase the risk of postoperative bacterial contamination, transient bacteremia, and surgical site infection, particularly in procedures involving manipulation of gingival tissues, periapical tissues, or oral mucosa [70]. Therefore, although routine antibiotic use is not justified in all oral surgical procedures, antibacterial protection may be clinically necessary in selected high-risk situations, especially when the surgical field is extensive, healing is compromised, or the patient presents with systemic risk factors [71].

This consideration is particularly relevant in medically compromised patients, including individuals receiving antiresorptive therapy such as bisphosphonates or denosumab. In these patients, invasive dentoalveolar surgery may increase the risk of medication-related osteonecrosis of the jaw, and careful infection control, atraumatic surgical technique, and optimized postoperative healing are essential elements of clinical management [72]. In such cases, locally delivered antimicrobial agents may offer a rational adjunctive approach by providing high drug concentrations at the surgical site while limiting systemic exposure.

**Figure 3:**
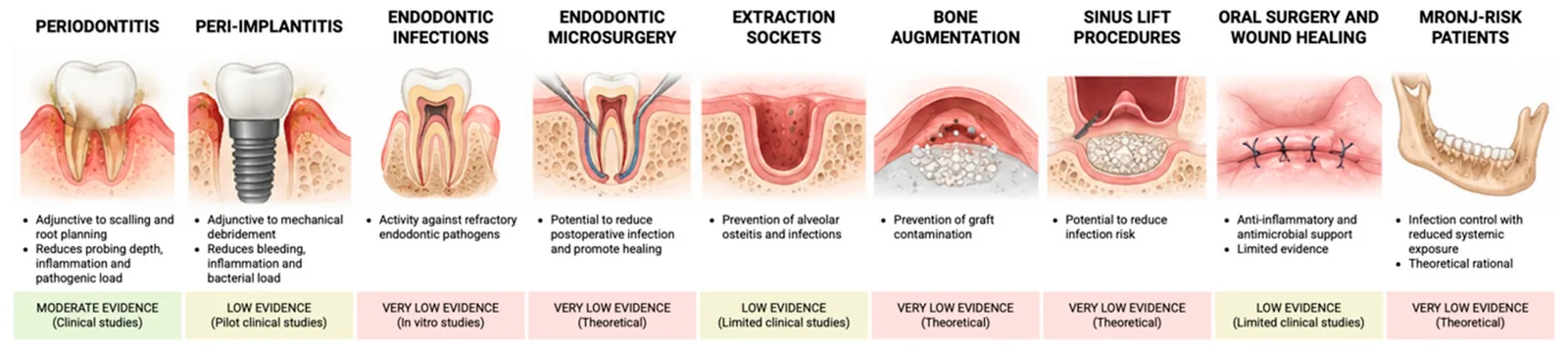
Potential dental applications and current level of evidence.

Azithromycin may be particularly interesting in this context because, beyond its antibacterial activity, it exhibits anti-inflammatory and immunomodulatory properties that may help control postoperative inflammation and promote tissue healing [42]. Clinical studies evaluating azithromycin as prophylaxis in oral surgery, including impacted third-molar surgery, suggest potential usefulness in reducing postoperative infectious complications (Table 2, Figure 3). However, the available evidence remains insufficient to recommend routine use [73]. Further studies are therefore required to determine whether local azithromycin delivery could improve outcomes in selected oral surgical and medically compromised patient populations [74].

## 6. Drug Delivery Systems for Local Application

**Figure 4:**
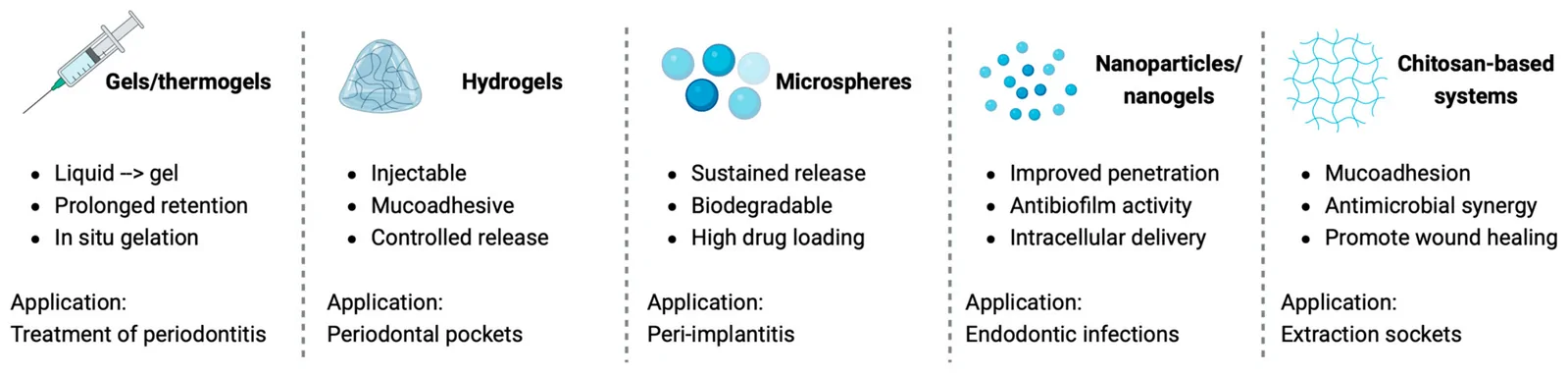
Local drug delivery systems for azithromycin in dentistry.

Local delivery platforms are designed to overcome key limitations of systemic antibiotic therapy in dentistry by (1) achieving high drug concentrations at the diseased site, (2) extending contact time in a saliva-rich environment, and (3) potentially reducing systemic exposure. For AZC, formulation development is particularly challenging because the drug has poor aqueous solubility and its therapeutic benefit may depend not only on antibacterial activity but also on anti-inflammatory/immunomodulatory effects, which may require sustained local presence. Recent work has therefore focused on delivery systems that improve solubility, enhance retention within periodontal pockets or wounds, and provide controlled release (Figure 4).

### 6.1. Subgingival Controlled-Release Gels (Periodontal Pocket Delivery)

Bioabsorbable gels represent the most clinically intuitive approach for AZC local delivery in periodontology: they can be placed directly into periodontal pockets after scaling and root planing (SRP), allowing site-specific exposure while minimizing systemic dosing [79].

Multiple clinical studies have evaluated 0.5% azithromycin gel as an adjunct to SRP in chronic periodontitis, reporting improvements in clinical and/or microbiological parameters compared with SRP alone (e.g., reductions in probing depth and inflammatory indices). Early clinical and microbiologic evidence for an indigenously prepared bioabsorbable, controlled-release 0.5% AZC gel was reported in [80].

Subsequent trials continued to explore this concept, including additional clinical evaluations of subgingival delivery of 0.5% AZC gel [81] and a randomized, placebo-controlled trial in smokers [77].

A later randomized clinical trial also investigated locally delivered 0.5% AZC as an adjunct in chronic periodontitis with type 2 diabetes, reflecting interest in medically compromised populations where limiting systemic antibiotic exposure may be desirable [82].

### 6.2. Thermoresponsive and Vesicular Systems

To improve pocket retention and controlled release, thermoresponsive systems (liquid at room temperature, gelling at body temperature) have been studied. A notable example is a thermoresponsive azithromycin-loaded niosome gel, developed specifically for intra-periodontal pocket delivery to improve sustained release and local efficacy in periodontitis-related settings [83].

### 6.3. Hydrogels and Solubility-Enhancing Approaches (Including Nano-Enabled Hydrogels)

Because AZC is poorly water-soluble, several formulation strategies aim to increase local bioavailability while preserving controlled release. A 2025 study described the optimization of azithromycin-based soft hydrogels and reported a nanoparticle-enabled approach to enhance AZC solubility and achieve controlled local release for dental applications [84].

### 6.4. Microspheres for Sustained Release (e.g., PLGA-Based Depots)

Biodegradable polymeric microspheres (often PLGA-based) are widely used for sustained drug delivery because they can provide prolonged release over days to weeks and reduce the need for repeated application [85].

A recent example directly relevant to dentistry/periodontology is azithromycin-loaded PLGA microspheres coated with silk fibroin, designed to reduce burst release and improve local anti-inflammatory effects; the authors reported beneficial outcomes in vivo, including attenuation of periodontal inflammation and recovery of periodontal tissue parameters in an experimental setting [86].

### 6.5. Nanoparticles and Nanogels (Anti-Biofilm Performance and Mucosal Compatibility)

Nanocarriers can enhance AZC performance by improving solubility, promoting penetration into biofilms, and enabling interaction with mucus and tissues. Although much of the mechanistic nanomedicine literature is not dental-specific, it is highly informative for oral biofilm-associated infections because the underlying challenge, namely dense and protective biofilm architecture, is shared across multiple clinical settings [87].

Importantly, nanoparticle- and hydrogel-based drug delivery systems are already extensively investigated in dentistry for the local administration of antimicrobial, anti-inflammatory, and regenerative agents. Various nanocarriers, including PLGA nanoparticles, liposomes, nanogels, chitosan-based systems, and electrospun nanofibers, have been evaluated for the delivery of chlorhexidine, doxycycline, minocycline, metronidazole, silver nanoparticles, and growth factors in periodontal, peri-implant, endodontic, and regenerative applications [88,89].

These systems demonstrated the ability to prolong drug retention, improve local bioavailability, enhance penetration into oral biofilms, and reduce systemic exposure, which are particularly important advantages in the oral environment characterized by saliva flow and difficult anatomical access. Consequently, the concept of locally delivered azithromycin nanoformulations does not represent an entirely new therapeutic paradigm, but rather an extension of already investigated dental drug-delivery technologies toward a molecule with combined antimicrobial and immunomodulatory activity.

For instance, nanoparticle platforms, including PLGA-based nanoparticles and nanogels, have already been shown to improve azithromycin’s antimicrobial and anti-biofilm effects and, in some systems, maintain low eukaryotic cytotoxicity while enhancing activity against established biofilms [90,91]. Moreover, recent dental-focused studies investigating azithromycin-loaded nanoparticles incorporated into chitosan-based hydrogels further support the feasibility of localized AZC delivery within oral tissues [84].

From a translational dentistry perspective, these findings support the plausibility of AZC nanoformulations for periodontal pockets, peri-implant tissues, extraction sites, or regenerative surgical procedures, where biofilm resilience and incomplete mechanical access remain major therapeutic barriers (Table 3).

## 7. Safety, Regulatory, and Industrial Translation Considerations

The local application of azithromycin in dentistry necessitates careful evaluation of safety, biocompatibility, and regulatory compliance before it can be incorporated into routine clinical practice. Although azithromycin has a well-established safety profile when administered systemically, local delivery introduces distinct biological and regulatory considerations that must be addressed independently.

One of the primary safety concerns with locally delivered antibiotics is their potential cytotoxicity to oral tissues. Periodontal ligament fibroblasts, gingival epithelial cells, osteoblasts, and peri-implant soft tissues may be exposed to drug concentrations significantly higher than those achieved systemically. While azithromycin is generally considered to have low cytotoxicity at therapeutically relevant concentrations, in vitro studies indicate that its effects may be dose- and exposure-time-dependent [93]. Therefore, formulation design must balance antimicrobial efficacy with cellular compatibility to avoid impairing wound healing or tissue regeneration. Biocompatibility testing of delivery vehicles, such as polymers, gels, or nanoparticles, is equally critical, as adverse tissue reactions may result from the carrier rather than the drug itself. Long-term exposure studies are particularly relevant for sustained-release systems that maintain drug presence in the oral cavity over extended periods.

The potential to induce antimicrobial resistance remains a major concern in both systemic and local antibiotic use [94]. Although local delivery reduces overall systemic exposure, it may still promote the selection of resistant microorganisms if subinhibitory concentrations persist at the treatment site [95]. This risk is particularly relevant for sustained-release formulations that exhibit an initial therapeutic peak followed by prolonged low-level exposure. From a stewardship perspective, the local use of azithromycin should be restricted to well-defined clinical indications and employed only as an adjunct to definitive mechanical or surgical therapy. Continuous monitoring of resistance patterns in the oral microbiota is essential to assess the long-term impact of local macrolide exposure, particularly given the increasing prevalence of macrolide-resistant bacterial strains worldwide.

Although systemic absorption following local application is expected to be minimal, it cannot be entirely excluded, particularly when large surface areas are treated or when the drug is applied repeatedly. Consequently, potential systemic effects, such as gastrointestinal intolerance or cardiac electrophysiological effects, should be considered, especially in medically compromised patients. Pharmacokinetic studies assessing systemic drug levels following local application are therefore an important component of safety evaluation.

At present, azithromycin is approved primarily for systemic use in a variety of infections [13,96], while dedicated formulations for local dental administration remain under investigation. Consequently, the local use of azithromycin in dentistry is an emerging, exploratory therapeutic approach that requires further validation through preclinical and clinical studies. Future development of locally delivered azithromycin formulations should include detailed toxicological assessment, stability evaluation, and well-designed randomized controlled trials to establish their safety, efficacy, and optimal clinical indications. Importantly, growing evidence suggests that AZC may exert biological effects that extend beyond antimicrobial activity alone. Experimental studies have demonstrated that azithromycin can inhibit human osteoclast function in vitro and reduce osteoclastic resorptive activity across different concentrations, potentially supporting bone preservation and regenerative healing processes [97]. These findings have encouraged consideration of topical azithromycin application in oral surgical procedures, particularly before soft-tissue closure, as an adjunct to conventional perioperative antibiotic strategies. Nevertheless, despite this growing experimental rationale, azithromycin is not currently specifically licensed for local dental applications, and most potential uses in dentistry would presently be considered off label. This regulatory status highlights the importance of careful case selection, informed consent, ethical oversight, and adherence to institutional and national guidelines when considering investigational or non-approved applications. For future regulatory approval, locally delivered azithromycin formulations will require rigorous preclinical and clinical evaluation, including toxicological testing, stability assessments, and well-designed randomized controlled trials demonstrating safety, biocompatibility, and clinical efficacy in oral tissues. Regulatory agencies may classify such products as medicinal products, combination devices, or drug–device combinations, depending on the delivery system employed, further complicating the approval pathway.

Given the current regulatory landscape, the local application of azithromycin should remain in clinical research until sufficient evidence supports its safety and efficacy. Standardized protocols, clearly defined dosing regimens, and long-term follow-up are necessary to ensure that potential benefits outweigh the risks. Importantly, safety considerations should be integrated into study design from the earliest stages of formulation development.

Beyond safety and regulatory considerations, successful clinical translation of locally delivered azithromycin formulations requires overcoming several manufacturing and industrialization challenges. Although numerous delivery systems, including hydrogels, nanoparticles, electrospun nanofibers, microspheres, and thermoresponsive formulations, have demonstrated promising preclinical performance, only a limited number have progressed toward commercial development.

One of the major challenges is ensuring batch-to-batch reproducibility while maintaining critical quality attributes, including particle size, drug loading, encapsulation efficiency, release kinetics, mechanical properties, and sterility. Scale-up of advanced delivery platforms frequently alters formulation characteristics because manufacturing parameters that are easily controlled at laboratory scale may become difficult to reproduce during industrial production [98,99].

Sterilization represents another important obstacle. Conventional techniques, including steam sterilization or gamma irradiation, may affect polymer integrity, alter azithromycin stability, or modify drug-release behavior. Therefore, sterilization procedures must be carefully optimized for each delivery platform.

Long-term storage stability also remains an important consideration. Many hydrogel- and nanoparticle-based formulations require protection against moisture, temperature fluctuations, aggregation, or premature drug degradation, underscoring the need for appropriate packaging and stability testing in accordance with ICH guidelines.

From a regulatory perspective, many local azithromycin formulations would likely be classified as combination drug-device products, requiring comprehensive evaluation of pharmaceutical quality, biocompatibility, manufacturing consistency, and clinical performance. Consequently, successful commercialization will require close collaboration among formulation scientists, clinicians, manufacturers, and regulatory authorities to ensure scalable, reproducible, and cost-effective production while maintaining safety and therapeutic efficacy [100].

## 8. Limitations of the Local Azithromycin Concept

Despite the promising pharmacological profile of azithromycin, several limitations should be acknowledged before considering its broader clinical application as a locally delivered therapeutic agent in dentistry.

The evidence base supporting local azithromycin delivery remains substantially smaller than that for established local antimicrobial agents such as doxycycline or minocycline [57]. Both tetracycline derivatives have been extensively investigated in periodontal therapy and are supported by commercially available delivery systems and long-term clinical data [101,102]. In contrast, most evidence regarding local azithromycin derives from small clinical studies, preclinical investigations, formulation-development research, or indirect extrapolation from systemic use (Table 4). Doxycycline, minocycline, and metronidazole were selected for comparison because they represent the most extensively studied and clinically established antibiotics for local delivery in dentistry. Commercial formulations of doxycycline and minocycline are widely used as adjuncts in periodontal therapy, whereas metronidazole has been extensively investigated in locally delivered gels and other formulations for the management of anaerobic oral infections. Their well-documented clinical efficacy, availability of commercial products, and inclusion in international periodontal treatment guidelines make them the most appropriate comparators for evaluating the translational potential of locally delivered azithromycin [103,104].

## 9. Future Directions and Research Gaps

Despite growing interest in the local application of azithromycin in dentistry, the current body of evidence remains fragmented and largely limited to small clinical trials, in vitro studies, and preclinical models. To define the role of locally delivered azithromycin in clinical practice, several key research gaps must be addressed through well-designed translational and clinical investigations.

Randomized controlled clinical trials with adequate sample sizes are essential for evaluating the efficacy and safety of locally applied azithromycin across various dental indications. Future studies should include standardized clinical endpoints, such as probing pocket depth reduction, clinical attachment level gain, stabilization of periodontitis and peri-implantitis progression, including limitation of further alveolar bone loss, inflammatory markers, and microbiological outcomes. Comparative trials assessing azithromycin against established local antimicrobials or antiseptics would be particularly valuable in determining its relative clinical benefit. Long-term follow-up is necessary to assess the durability of treatment effects and to monitor potential delayed adverse events.

Currently, the lack of extended observation periods limits conclusions regarding sustained efficacy and recurrence rates following local azithromycin therapy. However, the existence of approved topical ophthalmic azithromycin formulations, such as Azyter 15 mg/g eye drops, solution in single-dose containers, confirms that azithromycin can be formulated for local administration with acceptable topical tolerability in a non-systemic setting. Azyter contains azithromycin dihydrate at 15 mg/g and is formulated as a preservative-free oily solution based on medium-chain triglycerides, intended for short-course local antibacterial treatment of susceptible ocular infections [92]. Although ophthalmic use cannot be directly extrapolated to oral tissues, this existing formulation provides a useful translational reference point for evaluating several key limitations of local azithromycin delivery, including solubility, stability, tissue compatibility, local irritation, antimicrobial activity, and systemic exposure. In dentistry, such a formulation could be further investigated after incorporation into mucoadhesive or biodegradable carriers, such as chitosan-based hydrogels, to improve retention within periodontal pockets, extraction sockets, peri-implant defects, or regenerative surgical sites. This approach appears particularly promising because chitosan-based systems are already widely studied in oral drug delivery for their mucoadhesive, biocompatible, and antimicrobial properties. Therefore, combining an existing topical azithromycin formulation concept with chitosan-based delivery platforms may represent a rational first step toward developing dental-specific local AZC formulations. Nevertheless, dedicated in vitro and in vivo studies are required to assess compatibility with oral tissues, effects on dentin and bone cells, release kinetics, antimicrobial efficacy against oral biofilms, and safety under conditions typical of the oral cavity.

Optimal dosing regimens for local azithromycin delivery have not yet been established. Future research should focus on defining concentration ranges that maximize antimicrobial and immunomodulatory effects while minimizing cytotoxicity and the risk of resistance. Advanced delivery systems capable of controlled and predictable release profiles require further refinement and validation in clinically relevant models. In addition, studies should investigate the interaction between azithromycin formulations and the oral environment, including saliva, gingival crevicular fluid, and biofilms, to better understand drug stability and retention under real-world conditions.

The long-term impact of local azithromycin use on oral microbial ecology and antimicrobial resistance patterns remains poorly understood. Future investigations should include comprehensive microbiome analyses to evaluate shifts in microbial composition and the emergence of resistant strains following local therapy. This aspect is particularly important given the global rise in macrolide resistance and the oral cavity’s role as a reservoir for resistant microorganisms.

Personalized dental therapy represents an emerging paradigm in which treatment strategies are tailored to individual patient characteristics, including disease severity, microbiological profile, systemic health status, and genetic or immunological factors. Research exploring patient-specific predictors of response to local azithromycin therapy could support more targeted and effective use of this agent. For example, patients with aggressive inflammatory phenotypes or specific pathogenic profiles may derive greater benefit from azithromycin’s immunomodulatory effects.

Bridging the gap between experimental studies and clinical implementation requires coordinated translational research efforts. Preclinical models that more accurately replicate human oral disease, followed by phased clinical trials, are essential for establishing both efficacy and safety. Additionally, collaboration with regulatory authorities may facilitate the development of standardized evaluation frameworks for locally delivered antibiotics in dentistry.

Future research should prioritize well-designed randomized controlled clinical trials and translational studies to validate the encouraging findings obtained from in vitro and animal models before routine clinical implementation can be recommended.

## 10. Conclusions

Azithromycin is well established as a systemically administered antibiotic in dentistry, particularly for the management of odontogenic infections and as an alternative for patients with beta-lactam hypersensitivity. Beyond its antimicrobial activity, its distinctive pharmacokinetic profile and well-documented anti-inflammatory and immunomodulatory effects distinguish it from many other antibiotics used in dental practice. These properties are especially relevant in chronic inflammatory oral diseases, where tissue destruction results from a complex interplay between microbial challenge and host immune dysregulation.

The growing body of experimental and preliminary clinical evidence suggests that azithromycin may also hold promise as a locally delivered therapeutic agent in dentistry. Local application has the potential to achieve high drug concentrations at the site of disease, enhance anti-biofilm activity, and modulate local inflammatory responses, while simultaneously reducing systemic exposure and supporting antibiotic stewardship principles. These advantages are particularly appealing in periodontology, peri-implant therapy, endodontics, and selected oral surgical indications.

However, despite encouraging findings, the clinical use of locally delivered azithromycin remains investigational. Current evidence is limited by heterogeneity in study design, delivery systems, dosing regimens, and outcome measures. Critical issues, including long-term safety, effects on the oral microbiome, resistance development, and regulatory pathways, have yet to be adequately addressed. Consequently, routine clinical implementation cannot be recommended at this stage.

Future progress will depend on well-designed translational research that integrates formulation science, preclinical validation, and adequately powered randomized controlled clinical trials. Such efforts should aim to identify clearly defined indications, optimal delivery platforms, and patient populations most likely to benefit from this approach. If these challenges are successfully addressed, local azithromycin therapy may emerge as a valuable adjunctive strategy within modern, evidence-based, and stewardship-oriented dental care.

## Figures and Tables

**Table 1:** Why is azithromycin an attractive candidate for local delivery in dentistry.

Property	Clinical Relevance	Advantage of Local Delivery	Supporting Evidence	Level of Evidence
High tissue penetration	High concentrations in periodontal and peri-implant tissues	Sustained local drug availability	Human pharmacokinetic studies demonstrating high tissue/plasma ratios and GCF penetration [22,23]	Level 3
Intracellular accumulation	Activity against intracellular pathogens and persistence at inflammatory sites	Activity against intracellular pathogens and persistence at inflammatory sites	Pharmacokinetic and mechanistic studies [22]	Level 3
Anti-biofilm activity	Improved activity against mature oral biofilms	Improved activity against mature oral biofilms	In vitro biofilm studies [49]	Level 5
Immunomodulation	Reduction in inflammatory cytokines and tissue destruction	Reduction in inflammatory cytokines and tissue destruction	Experimental and mechanistic studies [38,39,42]	Level 5
Long half-life	Prolonged therapeutic effect	Prolonged therapeutic effect	Human pharmacokinetic studies [19,22]	Level 3
Broad antimicrobial spectrum	Activity against major oral pathogens	Activity against major oral pathogens	Microbiological and clinical studies [20,21,29]	Level 2–3

Levels of evidence: Level 1: Systematic reviews or meta-analyses of randomized controlled trials (RCTs), or high-quality diagnostic/prognosis studies. Level 2: Individual randomized controlled trials or cohort studies with robust follow-up. Level 3: Non-randomized controlled trials, case–control studies, or observational studies. Level 4: Case series or poor-quality observational studies. Level 5: Expert opinion, bench research, or first-principles physiological reasoning. Levels of evidence were adapted from the Oxford Centre for Evidence-Based Medicine (OCEBM) classification.

**Table 2:** Potential dental applications of locally delivered azithromycin and the current level of evidence.

Clinical Indication	Potential Mechanism	Available Evidence	Level of Evidence	Main Limitation
Periodontitis	Anti-biofilm + immunomodulation	Randomized clinical trials and systematic reviews [26,54,75,76,77]	Level 1–2	Limited number of studies; small sample sizes
Peri-implantitis	Biofilm suppression	Pilot clinical studies [78]	Level 3	Lack of large RCTs
Endodontic infections	Activity against refractory pathogens	In vitro [59]	Level 5	No clinical data
Endodontic microsurgery	Healing support	Theoretical rational	Level 5	No direct evidence
Bone augmentation	Infection prevention	Indirect clinical evidence [66]	Level 3	No studies evaluating local azithromycin
Sinus lift	Graft protection	Indirect clinical evidence [62]	Level 3	No studies evaluating local azithromycin
Oral surgery	Anti-inflammatory effects	Clinical studies of systemic azithromycin [68]	Level 2–3	Lack of studies on local delivery
MRONJ-risk patients	Infection control	Expert opinion and theoretical rationale	Level 5	No direct clinical evidence

Levels of evidence: Level 1: Systematic reviews or meta-analyses of randomized controlled trials (RCTs), or high-quality diagnostic/prognosis studies. Level 2: Individual randomized controlled trials or cohort studies with robust follow-up. Level 3: Non-randomized controlled trials, case-control studies, or observational studies. Level 4: Case series or poor-quality observational studies. Level 5: Expert opinion, bench research, or first-principles physiological reasoning. Levels of evidence were adapted from the Oxford Centre for Evidence-Based Medicine (OCEBM) classification.

**Table 3:** Drug delivery platforms for local azithromycin administration in dentistry.

Delivery System	Main Characteristics	Main Advantage	Main Advantage	References
Subgingival controlled-release gels	Direct placement into periodontal pockets; prolonged local exposure; minimal systemic absorption	Clinically tested	Limited retention	[52,77,80]
Thermoresponsive niosomal gels	Liquid during application, gelation at body temperature; improved retention and sustained release	Better retention	Experimental	[83]
Hydrogels	Controlled release, biocompatibility, potential mucoadhesion	Mucoadhesion	Limited clinical validation	[84]
Chitosan-based hydrogels	Mucoadhesive properties, enhanced retention, biocompatibility, possible synergistic antimicrobial effects	Mucoadhesion, antimicrobial activity	Experimental	[45,84]
PLGA microspheres	Sustained release over days to weeks; reduced need for repeated administration	Sustained release	Manufacturing complexity	[85,86]
PLGA nanoparticles	Improved biofilm penetration; enhanced intracellular delivery; controlled release	Biofilm penetration	Regulatory complexity	[90]
Nanogels	Improved solubility and anti-biofilm activity; low cytotoxicity	Low cytotoxicity	Limited oral data	[91]
Liposomes and vesicular systems	Enhanced tissue interaction and drug protection; potential controlled release	Enhanced tissue interaction	Stability issues	[88]
Electrospun nanofibers	High surface area, prolonged local drug release, scaffold function	Scaffold + drug delivery	No AZC-specific studies	[88]
Repurposed ophthalmic AZM formulations (e.g., Azyter-based concept)	Existing approved local AZM formulation; may serve as a starting point for dental-specific formulations	Translational potential	Not optimised for oral use	[92]

**Table 4:** Comparison of azithromycin with established locally delivered antimicrobial agents used in dentistry.

Feature	Azithromycin	Doxycycline	Minocycline	Metronidazole
Antimicrobial activity against periodontal pathogens	High	High	High	Mainly anaerobic bacteria
Anti-biofilm activity	High	Moderate-High	Moderate-High	Moderate
Immunomodulatory activity	Strongly documented	Documented (MMP inhibition)	Limited	Minimal
Intracellular accumulation	High	Moderate	Moderate	Low
Tissue penetration	High	Moderate	Moderate	Moderate
Evidence for local dental delivery	Limited	Extensive	Extensive	Moderate
Commercial local formulations available	No	Yes	Yes	Limited
Potential role in peri-implantitis	Emerging	Established adjunctive use	Established adjunctive use	Limited evidence
Potential role in regenerative procedures	Theoretical/experimental	Limited	Limited	Minimal
Main limitation	Limited clinical evidence	Risk of resistance and staining	Tooth discoloration, resistance	Narrow antimicrobial spectrum

## Data Availability

No new data were created or analyzed in this study. Data sharing is not applicable to this article.
